# Unmasking the Intruder: A Case of Lung Carcinoma Mimicking As Glioma

**DOI:** 10.7759/cureus.68035

**Published:** 2024-08-28

**Authors:** Joana Christopher, Thanka Johnson, Anbukkarasi Kannan, Santhanam Rengarajan

**Affiliations:** 1 Pathology, Sree Balaji Medical College and Hospital, Chennai, IND; 2 Neurosurgery, Sree Balaji Medical College and Hospital, Chennai, IND

**Keywords:** oncology, neurosurgery, histopathology, brain metastases, lung carcinoma

## Abstract

Lung carcinoma with metastases to the brain typically involves the cerebral hemispheres, occasionally the cerebellum and rarely the brainstem. This report presents the case of a 73-year-old male who complained of neurological symptoms of left-sided limb weakness and was initially diagnosed with a high-grade glioma. With further radiological investigations, our patient was found to have primary bronchogenic carcinoma with metastasis to the brain. He underwent craniotomy with decompression of the mass and is currently undergoing chemotherapy. This case calls for the collaborative effort needed to diagnose brain metastases and the importance of histopathology for confirmation of the same.

## Introduction

The brain is the most crucial component of our physiology, as it governs and processes the majority of our bodily functions. This is why high-grade primary tumors or metastases to the brain drastically decrease the survival rate of most that are inflicted. The majority of intra-cranial tumors are a result of metastases with lung cancer, accounting for a large proportion of these tumors. Despite the advancement in treatment for brain tumors, those with lung metastases to the brain continue to have a dismal survival rate. This has led to the usage of prophylactic cranial irradiation when a diagnosis of lung cancer is made [1]. Lung cancers are broadly classified as non-small cell lung cancer and small cell lung cancer, with 10% of the patients with small cell lung carcinoma manifesting with brain metastases during their first visit to the hospital [2-3]. We present a case of incidental lung adenocarcinoma, initially thought to be a primary central nervous system tumor, as the patient had only reported weakness and numbness of limbs. Later on, with more probing and radiology, we found our patient to be a case of primary bronchogenic carcinoma with metastasis to the brain mimicking a high-grade glioma. Our patient is being managed by craniotomy with decompression of the frontal mass and chemotherapy.

## Case presentation

A 73-year-old male patient presented to our institute complaining of acute left-sided upper limb and lower limb weakness for three weeks. He also presented with numbness in the left half of his body. He was dull in appearance and slow to respond with complaints of giddiness over a span of three weeks. He complained of finding it difficult to walk and swallow his food. He lost his appetite three months ago and noticed he had lost 10 kilograms of weight in six months. He had no other complaints, like seizures or headaches. On further probing, our patient complained of a dry cough lasting just one week.

He indicated that he had previously been a smoker and had consumed alcohol regularly for over 20 years. However, he has maintained sobriety and abstained from nicotine for the past 15 years. The patient is a known case of hypertension and benign prostatic hyperplasia on regular treatment for both. On physical examination, the patient was oriented, obeyed commands, and had mild left-sided upper motor neuron type of facial weakness. The treating surgeon found left hemiparesis (grade 3/5) with plantar extension of the left foot. A CT scan of the brain using non-ionic intravenous contrast and an MRI of the brain were performed. A CT scan of the brain revealed a 51 × 50 x 47 mm well-defined irregular-shaped heterogenous mass in the right high frontal lobe.

**Figure 1:**
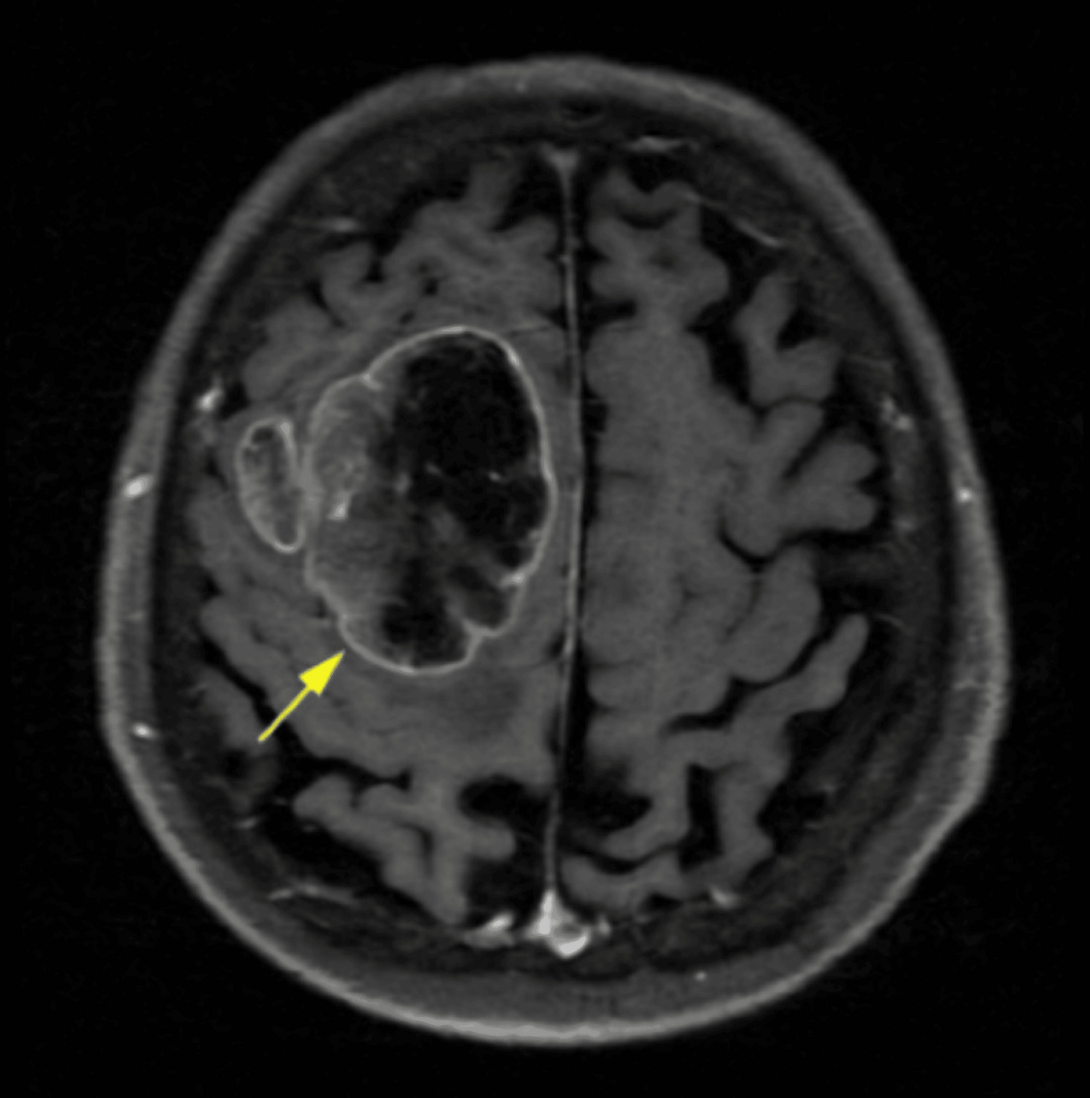
MRI of the brain T2 FLAIR image showing heterogenous solid cystic mass in the right frontal lobe (arrow) MRI: magnetic resonance imaging, FLAIR: fluid-attenuated inversion recovery

**Figure 2:**
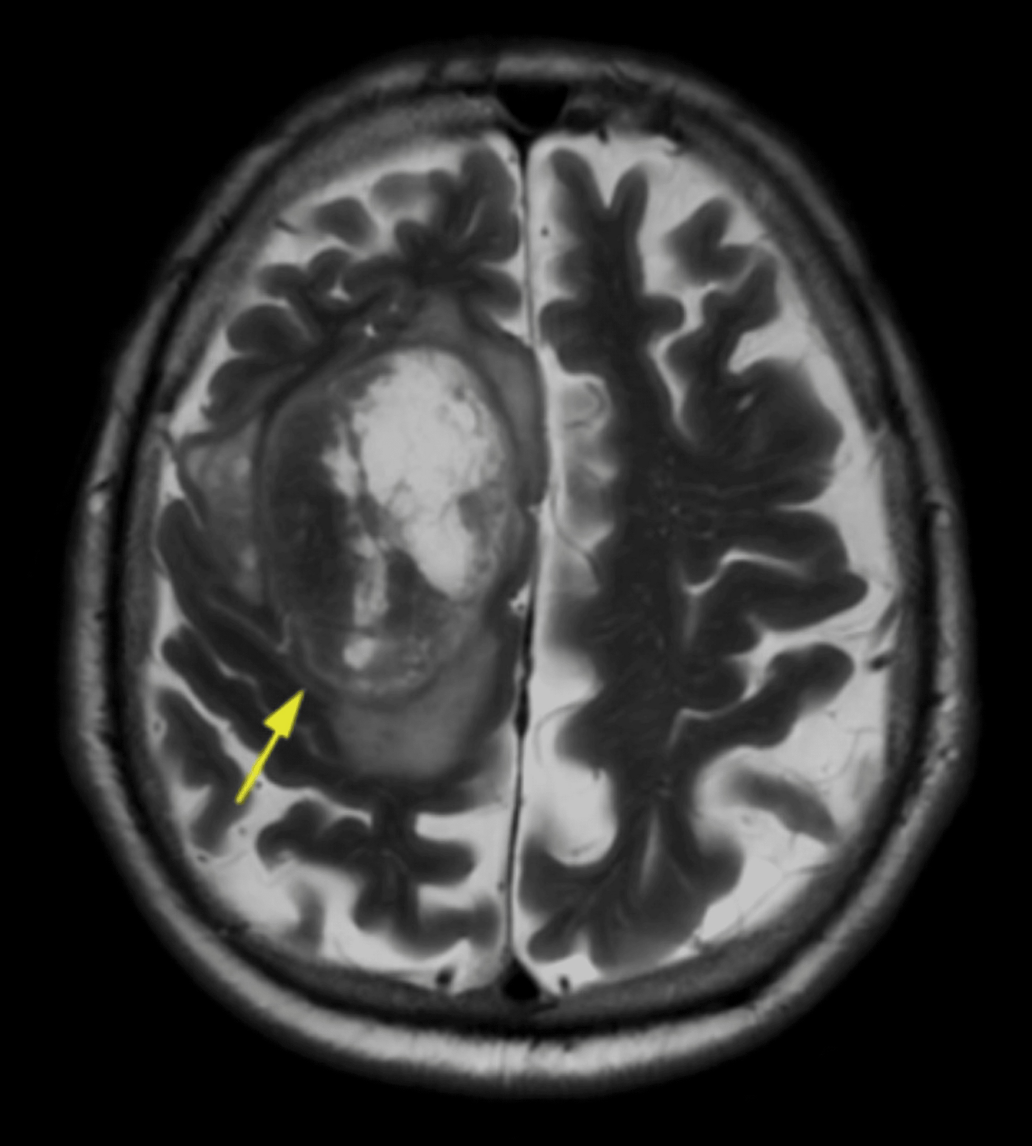
MRI of the brain showing heterogenous solid cystic mass in the right frontal lobe (arrow) MRI: magnetic resonance imaging

The mass showed heterogenous enhancement with an eccentric solid area along a superior aspect with large areas of necrosis. The solid areas revealed a few small curvilinear calcifications and mild perilesional edema, resulting in a mass effect causing focal midline shift of the falx. Ventricles, cisterns, and sulcal spaces were prominent with a persistent cavum septum pellucidum (normal variant) and diffuse age-related cerebral atrophy. The findings suggested the lesion was likely a glioma. A CT scan of the spine was also done and showed cervical spondylosis and left C4/C5 and C5/C6 facetal arthrosis. MRI of the brain with contrast and spectroscopy showed an ill-defined heterogenous solid cystic mass lesion measuring ~ 5.6 x 5.5 x 5.0 cm in the parasagittal aspect of the right frontal lobe involving the cortex, subcortical white matter, and deep white matter (Figures 1-2).

**Figure 3:**
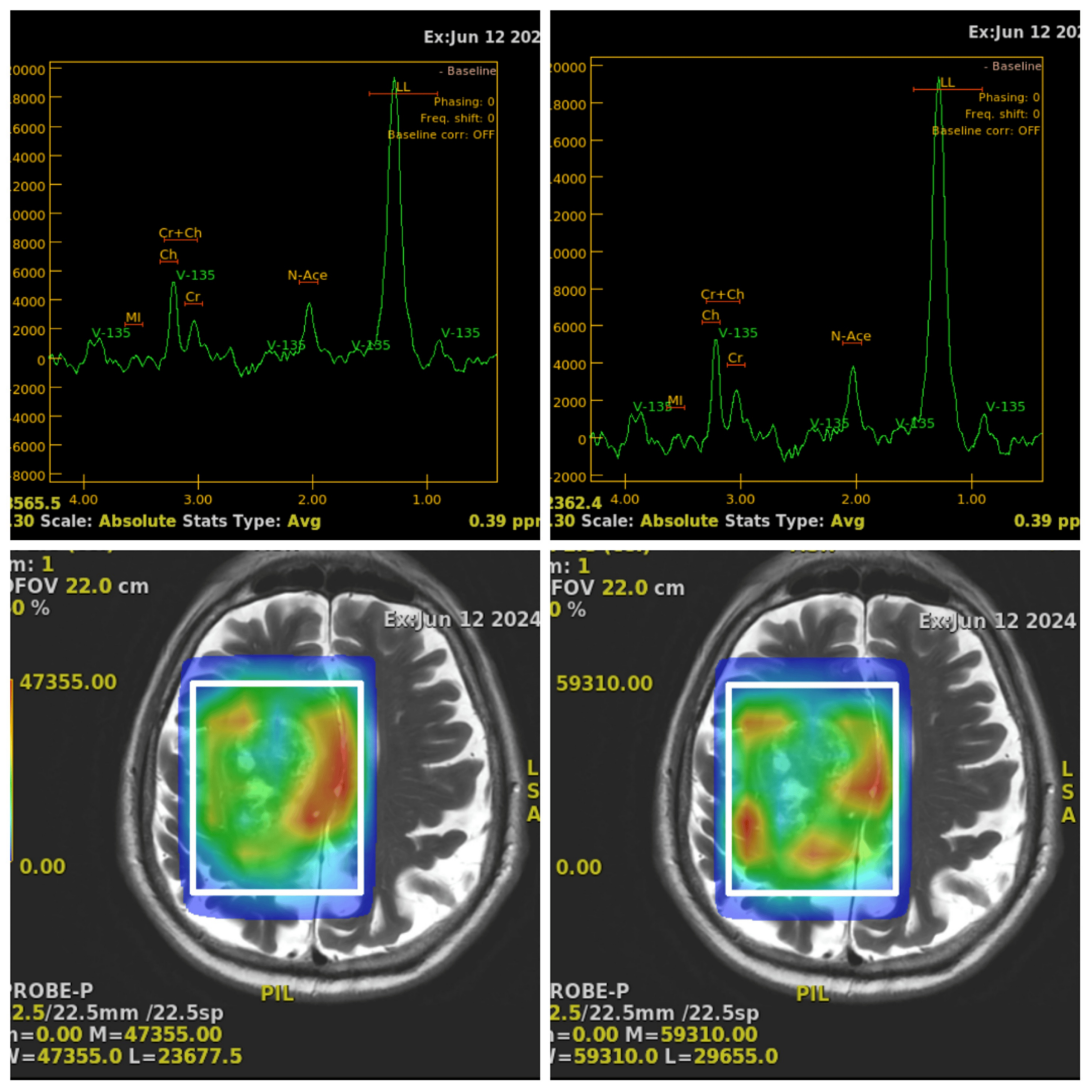
MR spectroscopy images of the tumor showing a moderate increase in the choline-creatinine ratio and a moderate decrease in N-acetylaspartate MR: magnetic resonance

The margins of the mass were irregular with a lobulated contour and showed a heterogenous enhancement with peripheral enhancing areas to central non-enhancing areas. There was significant perilesional edema noted involving the adjacent white matter. The lesion is seen exerting a mass effect and causing a midline shift of approximately 4.8 mm to the left side with a mild mass effect causing compression of the body of the right lateral ventricle. On MR spectroscopy, the solid enhancing area shows a moderate increase in the choline-creatinine ratio and a moderate decrease in N-acetylaspartate (Figure 3).

The above features suggested the lesion could likely represent a high-grade neoplastic etiology with a differential diagnosis of either glioblastoma multiforme or secondary metastasis. With the above features, a provisional diagnosis of right-sided posterior frontal mass - likely a high-grade glioma - was made. Further workup was done to check the surgical fitness status of the patient when a right hilar mass lesion was found on a chest X-ray incidentally with the possibility of being malignant, after which the patient underwent right frontal craniotomy with decompression of posterior frontal mass.

**Figure 4:**
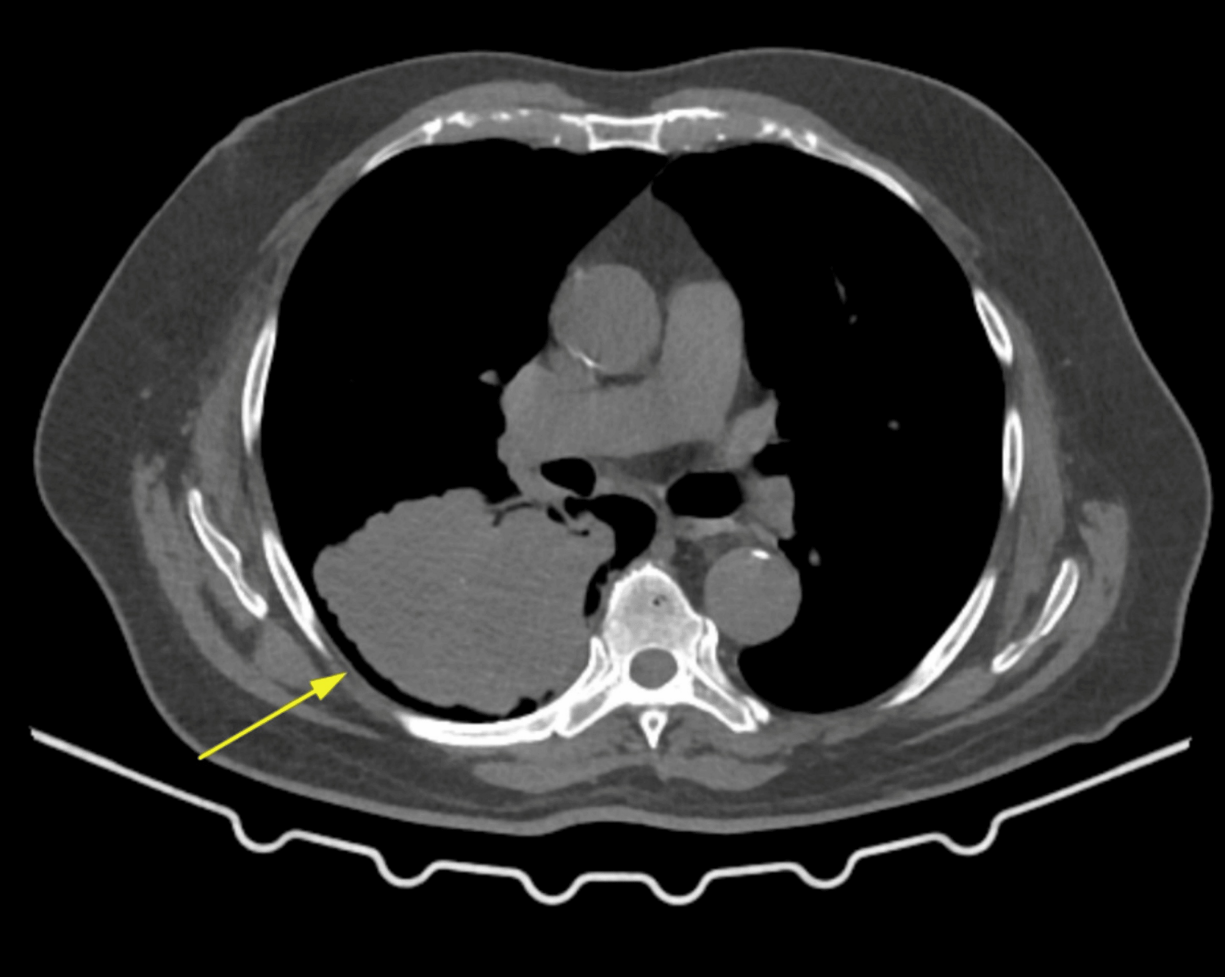
Contrast-enhanced CT of the chest showing a heterogenous lobulated mass in the right lower lobe of the lung (arrow) CT: computed tomography

A contrast-enhanced CT of the chest was then performed and found an ill-defined lobulated heterogenous space occupying a mass lesion measuring ~ 8.2 x 8.1 x 9.8 cm seen in the right lower lobe, causing an abrupt cut-off of the right lower lobe segmental bronchus. The mass is seen predominantly in the superior basal segment of the right lower lobe (Figure 4).

**Figure 5:**
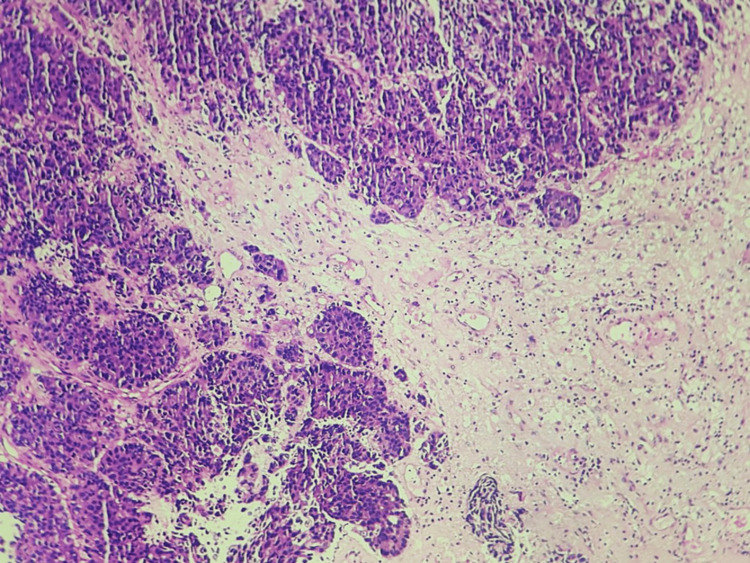
Microscopic picture of tumor cells arranged in nests and glandular pattern (100x, H&E) H&E: hematoxylin and eosin

It causes adjacent pleural tethering. The mass superiorly involves an oblique fissure and the external surface of the posterior segment of the right upper lobe. A pulmonary nodule measuring ~ 3.0 mm was seen in the lateral segment of the right middle lobe, and mediastinal lymphadenopathy was also noted. All the above features suggested this could be a mass of neoplastic etiology. The clinical diagnosis for our patient now included the brain lesion being either a high-grade glioma or a primary bronchogenic carcinoma with metastasis to the brain. The patient underwent a right frontal craniotomy with decompression of a posterior frontal mass to address his symptoms, resulting in favorable postoperative outcomes. Grossly, the tissue was seen as gray-brown fragments. On microscopic examination, an extremely tiny fragment of the cerebrum was observed with tumor deposits forming nests and glands of cells (Figure 5).

**Figure 6:**
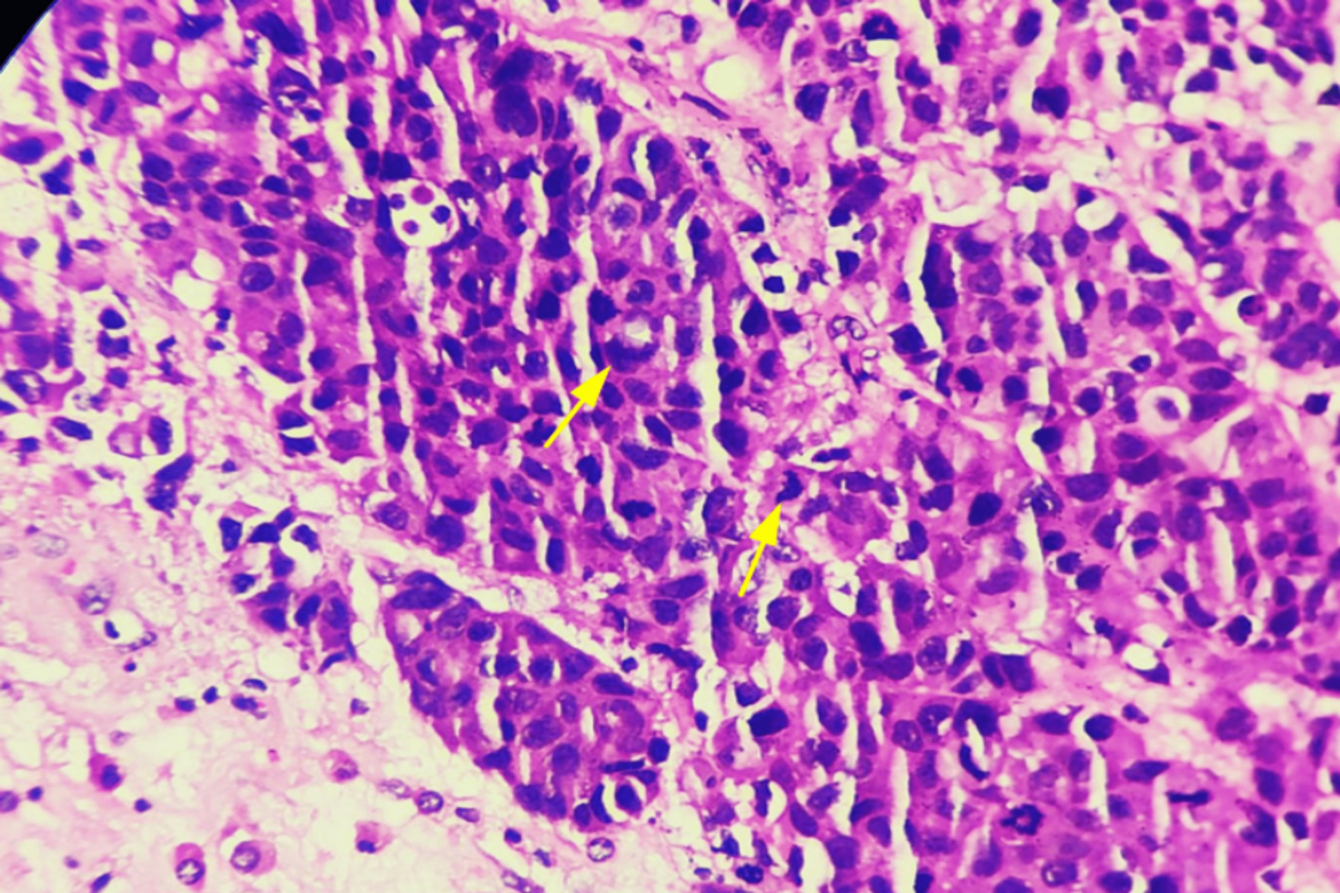
Microscopic picture showing tumor cells with features of nuclear pleomorphism, hyperchromatic nuclei, increased mitosis, and abnormal mitosis (400x, H&E). Arrows showing mitosis. H&E: hematoxylin and eosin

Individual tumor cells are seen showing hyperchromatic nuclei, nuclear pleomorphism, increased mitosis, and also abnormal mitosis (Figure 6).

**Figure 7:**
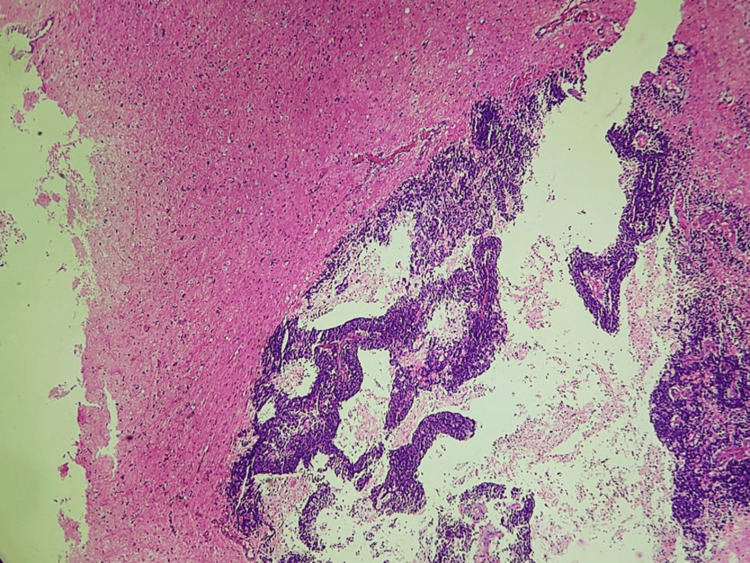
Microscopic picture showing a large area of necrosis around the tumor cells (100x, H&E) H&E: hematoxylin and eosin

Large areas of necrosis (30%) were also noted, with the adjacent brain tissue appearing normal (Figure 7).

The histopathological examination concluded that this may represent a metastatic tumor deposit in the brain of the primary bronchogenic carcinoma. The patient received adequate post-surgical care, psychological counseling, and physiotherapy and responded well to the treatment. He was referred to a higher cancer center for chemotherapy with a suggestion from the histopathology team for initial immuno-histochemical markers, which included thyroid transcription factor 1 (TTF-1), glial fibrillary acidic protein (GFAP), and cytokeratin for further categorization of the tumor. The patient is expected for his follow-up consultation at our institute post-chemotherapy.

## Discussion

Lung carcinoma and brain metastases

Lung cancer is the leading cause of death in the world, with 45-5-% of lung cancers metastasizing to the brain. Therefore, understanding the mechanisms revolving around this must be looked into [4]. Wang et al. studied 335 primary lung cancer patients with metastases to the brain and estimated the distribution of these lesions as follows: 21% of cases had a single lesion, 47% of the lesions were in the right frontal lobe, and 48% of these lesions were metastases from a lung adenocarcinoma [1]. According to previous studies and literature, there is a relationship between brain anatomy and brain metastasis; the cerebellum, gray-white matter junction, and watershed areas are preferentially involved. Paget’s theory or seed-soil theory, and the other possible explanation could be high blood perfusion in these areas.

The molecular pathway involved in lung cancer metastasis to the brain includes certain gene expressions that were studied using polymerase chain reaction and were found to increase invasion, adhesion, angiogenesis, and cell migration of tumor cells. These genes are kinesin motor (KIFC1), FALZ, N-cadherin (CDH2), and defective in the cullin neddylation 1 domain containing 1 (DCUN1D1), which relates to a poor prognosis [5-9]. The integrin α3β1 is proposed to be involved in brain metastases as it interacts with laminin to cause tumor cell migration and invasion [10]. Certain cell adhesion molecules and cadherins are also involved in metastases by increasing the tumor cell motility. Loss of e-cadherin expression is the cause for this increase in cellular motility. According to Yoo et al., metastatic brain tumors show increased e-cadherin levels, while low levels of e-cadherin expression in non-small cell lung carcinoma corresponded with increased risk for brain metastases [11]. Studies show that increased vascular endothelial growth factor (VEGF) and specific chemokines like C-X-C chemokine receptor type 4/C-X-C motif chemokine ligand 12 (CXCR4/CXCL12), epidermal growth factor receptor (EGFR) mutations, specific single nucleotide polymorphisms (SNPs), and increase carcinoembryonic antigen (CEA) tumor marker in non-small cell lung cancer and placental growth factor and increased pro-gastrin-releasing peptide tumor marker in small cell lung cancer, matrix metalloproteinase-9 (MMP-9) in lung adenocarcinoma cells, ADAM metallopeptidase domain 9 (ADAM9) overexpression, lymphoid enhancer-binding factor 1 (LEF1) overexpression, homeobox B9 (HOXB9) overexpression, expression of cellular mesenchymal-epithelial transition (c-Met) by lung cancer cells were involved in increasing the risk of brain metastases from lung carcinoma [4].

Brain metastases

Metastases to the brain can occur either through hematogenous spread or rarely through direct spread from nearby structures. The majority of brain metastases occur in the cerebral hemispheres, accounting for 80%, while 15% of metastases are found in the cerebellum and only 5% in the brainstem. Neurological symptoms like headaches, visual disturbances, altered mental status, ataxia, and sensory disturbances can occur. Twenty-five percent of patients who die of cancer die due to metastases to the brain. Brain metastases are seen more commonly in adults and rarely in the pediatric population. Lung carcinoma accounts for the highest frequency of primary tumors metastasizing to the brain. However, in 10% of the cases, the primary is never found. Grossly, they are seen as a circumscribed, grey-white to tan mass. Necrosis may be found at the center of the mass with edema around the lesion. Hemorrhage may or may not be seen depending on the primary tumor. A microscopic picture is also dependent on the primary tumor; small cell lung carcinomas and lymphomas can show diffuse infiltration and normal brain parenchyma is seen as a small focus in the periphery or surrounding the blood vessels. Detection of tumor cells not of central nervous system origin and the use of immunohistochemical markers are used for diagnosis of the primary tumor [12].

## Conclusions

This case is presented to show the importance of collaborative effort among various departments in diagnosing metastatic brain tumors. The brain tumor in our patient was first thought to be of primary origin. The initial high-grade glioma was later found to be a primary bronchogenic carcinoma with metastasis to the brain using specific radiological imaging and histopathological examination.

We conclude this case report with a note that, although patient history is important, it only points us in the right direction. As physicians, we must learn to discuss certain cases and their clinical details with various departments like radiology and histopathology, which will lead to a diagnosis sooner with this collaborative effort. Regular inter-departmental meetings can be held to learn the importance of the involvement of varied departments.
